# The Gynæcological Society of Chicago—Stated Meeting March 23

**Published:** 1888-06

**Authors:** 


### THE GYNÆCOLOGICAL SOCIETY OF CHICAGO.

Regular Meeting, March 23, 1888.

The President, Henry T. Byford, in the chair.

The President exhibited two uteri, removed per vaginam for fibro-sarcoma and carcinoma respectively. The patients were aged 47 and 55 years, and have both done well since the removal.

Dr. Franklin H. Martin read a report of fifteen cases of fibroid tumors of the uterus treated by galvanism. From January 1, 1887, to January 1, 1888, he applied galvanism in strong, accurately measured, and definitely concentrated doses in gynæcological cases over fourteen hundred times. During this time he employed galvanism for uterine fibroids 623 times in fifteen cases. The results were as follows:

| | |
|---|---|
| Not suitable for treatment, and recommended for operation | 1 |
| Benefited | 4 |
| Symptomatically cured | 5 |
| Absolutely cured | 5 |

The author of the paper then selected and gave in detail the history and treatment of five cases, two of which were symptomatic cures, two actual cures, and the remaining case was benefited. He then reported in detail the fifteen cases already referred to. The strength of the current was usually 100 milliamperes, and was passed for five minutes at each sitting. The abdominal electrode was the large membraneous one of his own devising, already described in this journal. The intra-uterine electrode was also of his own design. He employed the positive pole in the uterus to control menorrhagia by reason of its causti ceffects, but at other times the negative.

Dr. J. C. Hoag, read a paper, in which he discussed some considerations regarding death of the fœtus in utero.

A careful inquiry concerning the comparative frequency of premature expulsion of the impregnated human ovum can scarcely fail to surprise the physician, although anything like statistical accuracy in its determination is, for very obvious reasons, an utter impossibility.

Good authorities are of the opinion that we may safely reckon one case of abortion in the first months of pregnancy to every eight or ten cases of parturition at full term. Accordingly, the importance of careful study, to ascertain as fully as possible the proximate causes of this occurrence, cannot be overestimated.

As an example of the unsatisfactory state of our knowlege in this direction, one need only turn to the reports of large lying-in hospitals. In an interesting analysis of two years' work in Professor Gustave Braun's clinic, 6,230 labor cases are reviewed. Premature labor occurred in 565 cases, the causes being tabulated as follows:

| | |
|---|---|
| Syphilis (about) | 70 |
| Pulmonary tuberculosis | 9 |
| Peritonitis | 2 |
| Other fevers | 23 |
| Faulty placental insertion | 10 |
| Vitium cordis | 1 |
| Injuries | 7 |
| Eclampsia | 1 |
| Induction of labor | 4 |
| Twin pregnancy | 43 |
| Total | 170 |

This leaves 395 cases to be relegated to the category of the unknown.

In Braun, Chiari, and Spaeth's analyses of 7,835 cases of labor occurring in the Vienna Hospital for the year 1850–51, 393 cases of premature labor are included. In only 126 cases, or one-third, could the cause be definitely determined.

CAUSES OF THE PREMATURE INTERRUPTION OF PREGNANCY

These may be considered as they relate, first, to the father; second, to the mother; third, to the ovum; and fourth to traumatism.

*Causes Referable to the Father.*—These are constitutional vices, particularly those of syphilitic origin.

*Causes Referable to the Mother.*—These include general and local diseases, climatic and hygienic influences, and finally certain idiosyncrasies, which seem to be productive of abnormal irritability on the part of the uterus, even in the absence of recognizable lesions.

*Causes Referable to the Ovum.* — These comprise all diseased conditions of the fœtus and its appendages.

Traumatism includes accidents, efforts at criminal abortion, and the justifiable induction of labor.

In considering the subject of abortion in general, one can scarcely fail to be struck by the disproportionate frequency in multiparæ, and this seems clearly to depend on local diseases, such as endometritis, metritis, and uterine displacements.

If we exclude attempts at criminal abortion and the justifiable induction of labor, traumatism may be said to include only a small proportion of the causes of abortion; thus, in the two analyses above referred to, we find only fifteen cases referable to injuries in a total of 904 cases.

But whatever may be the mediate causes of the interruption of pregnancy, the death of the ovum is almost without exception the immediate cause of the awakened uterine activity and the premature expulsion of the product of conception.

Death of the fœtus may be determined by disturbances of nutrition, due to faulty development of the ovum, and especially its appendages, which lead to abstraction from the fœtus of its supply of nutriment and oxygen.

Again, the exchanges between fœtal and maternal blood may be impaired by maternal hæmorrhages, whether from the uterus or from other organs.

As for syphilis, this may cause the death of the fœtus, either primarily or secondarily.

The point of this paper is to draw attention to the influence of endometritis in determining the premature expulsion of the ovum. The great frequency of this disease is a matter of daily observation, and its influence in the production of abortion cannot be doubted.

If a woman aborts frequently, we are apt to say she aborts because she has aborted, she is the subject of habitual abortion. With this we content ourselves, whereas, no doubt, a careful study of these cases would often lead to the discovery of a pathological cause of remedial nature.

In endometritis, extravasations into the hypertrophied tissue of the decidua often take place. If the disease is of moderate extent, or if it develops late in pregnancy, and does not involve the placenta itself, it may be borne without influence upon the development of the fœtus. If, however, it develops earlier or in a severer form, it may often compromise the life of the fœtus, by leading to endometritis decidua with its hyperplasia, extravasations, and fatty infiltrations. If the placental decidua be involved, labor, when it comes on, is apt to be complicated by adherent placenta with its consecutive dangers.

*Report of Case.*—He here offered for inspection a specimen of macerated fœtus with its appendages. The patient who gave it birth was born in this State. She is 24 years of age, well developed, weighing about one hundred and sixty pounds, is strong, and claims to have enjoyed very excellent health for the most part. She was married three years ago, her husband being a skilled mechanic of exemplary habits and enjoying fairly good health.

Seven months after marriage, the patient gave birth to a macerated fœtus of six or seven months' development. One year later, this occurrence was duplicated. About a year and a half later still, she gave birth to the fœtus which was exhibited.

The placenta of the first fœtus was somewhat adherent, and did not come away entire. The patient spent two weeks in bed, and, according to her own statement, was pretty sick. Subsequent to this, she enjoyed good health until after the birth of the second fœtus, since which time she has not felt as well as before marriage.

A leucorrhœal discharge made its appearance after the birth of the second fœtus, and has continued most of the time to the present day, and since this event her menstrual periods have been accompanied by pain, headache, and fever. The patient has never flowed much during labor, and never at all during gestation.

On the 28th day of last December the patient said that she had reason to suspect that pregnancy had been interrupted, and requested an examination.

The examination revealed a doughy condition of the belly, which was not distended in proportion to the supposed period of gestation. The position of the fœtal parts could not be made out. The os externum admitted one finger. A thick muco-purulent discharge of disagreeable odor was present in considerable quantity.

As regards subjective sensations, the patient said that the last fœtal movements had been felt four days before, and that they had grown gradually feebler before altogether ceasing. She had also experienced sharp pains in the abdomen, and had noticed a feeling as though the fœtus had turned over, such as is often described in connection with a dead fœtus. She had not, however, noticed the other sensations of mawkish taste in the mouth, languor, and a sensation of cold in the abdomen from stoppage of the fœtal circulation, and concomitant abstraction of heat from the uterus—a phenomenon, by the way, which has been made available as a diagnostic point in determining whether the fœtus has died or not. The patient was advised that she might expect the expulsion of the fœtus in about ten days; requested to use the vaginal douche.

On the 5th day of January, twelve days after the cessation of fœtal movements, labor began. The fœtus was discharged, with the membranes intact, the placenta soon following under the influence of gentle compression of the uterus. The placenta was intact, and labor ended with a moderate discharge of blood.

With the exception of the presence of a small fresh coagulum on the surface of the placenta, a pale color, and considerable friability of its tissue, nothing abnormal in its appearance could be discerned.

The fœtus corresponds to the sixth month of development. It exhibits the usual appearances of maceration.

At noon of the sixth day of the puerperium the patient had a chill, and some additional severity in the pains from uterine contractions.

The temperature, which up to this time had been nearly normal, was now found to measure 100.5°. The lochia was somewhat fœtid, but the patient said she had passed no clots whatever. A vaginal examination revealed a subinvoluted uterus.

At 5 o'clock p. m., the temperature was 101.5°, and the uterus was curetted with a modified Sims' curette, and a quantity of soft pulpy material, composed almost wholly of fibrin and blood corpuscles, was removed; the total bulk of this material was about equal to the volume of a three-ounce vial.

At first I was unable to reach the fundus uteri with the instrument, but after repeated efforts, which resulted in bringing away consistent masses half as large as one's finger, I was able to reach every part of the endometrium. Every stroke of the curette dislodged masses of fibrin, but even after a most vigorous scraping the endometrium at the fundus had a rough, knobby feel.

The following morning, the patient, after a good night's rest, had a normal temperature and felt far more comfortable than at any time since her labor.

The temperature remained normal for three days, when it exhibited a considerable exacerbation, and the uterus was douched again. The speculum was necessary, because it was impossible to introduce the nozzle without it.

In this case, neither the patient nor her husband has ever been the subject of syphilis. The patient herself has undoubtedly long suffered from endometritis, although this was perhaps not the occasion of her first mishap.

Dr. Hoag further said, the more I use the curette in puerperal cases, the better pleased I am with it. I prefer an instrument with a large scraping surface and with a long shaft, set in a convenient handle. The presence of foreign bodies in the puerperal uterus is sure to obstruct the progress of involution, and the depth of the canal is altogether disproportionate to the size of the retained decidual or placental tissue or coagulum. Duncan has reported a case where a small bit of placenta was retained in which, at the time of the removal of the tissue eight months after the expulsion of the fetus, the uterine canal was eight inches deep. In my case, I found that involution had made no progress at the time of the curetting, and the instrument which I used, although eleven inches long, was almost too short.

I cannot help feeling that the employment of the finger-nail as a curette is a dangerous procedure. It is of interest in this connection to note the results of some recent experiments by Professor Fürbringer, as reported in the *N. Y. Med. Record* of March 10. The experimenter, employing for his subjects thirteen assistants and chiefs of clinics, required them to carefully disinfect their hands according to the most approved methods. He then succeeded in extracting enough septic material from under their finger-nails to start colonies in twelve out of the thirteen cases.

